# Influence of coronavirus disease 2019 on myopic progression in children treated with low-concentration atropine

**DOI:** 10.1371/journal.pone.0257480

**Published:** 2021-09-14

**Authors:** Hae Ri Yum, Shin Hae Park, Sun Young Shin

**Affiliations:** 1 Department of Ophthalmology and Visual Science, Eunpyeong St. Mary’s Hospital, College of Medicine, The Catholic University of Korea, Seoul, Republic of Korea; 2 Department of Ophthalmology and Visual Science, Seoul St. Mary’s Hospital, College of Medicine, The Catholic University of Korea, Seoul, Republic of Korea; Saarland University, GERMANY

## Abstract

**Purpose:**

The outbreak of coronavirus disease 2019 (COVID-19) has caused many children to stay indoors. Increased near work and insufficient outdoor activities are considered important risk factors for myopic progression. This study aimed to compare the changes in myopic progression before and after COVID-19 in children treated with low-concentration atropine.

**Methods:**

The records of 103 eyes of 103 children who were treated with low-concentration atropine eye drops were retrospectively reviewed. We classified children according to the concentration of atropine eye drops and children’s age. The beginning of the pre-COVID-19 period was set from January 2019 to May 2019, and the endpoint was set in March 2020. The beginning of the post-COVID-19 period was set in March 2020, and the endpoint was set from January 2021 to March 2021. We evaluated the questionnaires administered to children’s parents.

**Results:**

A significant myopic progression was observed in the post-COVID-19 period compared to the pre-COVID-19 period in the 0.05% and 0.025% atropine groups (P < 0.001 and P = 0.020, respectively). For children aged 5 to 7 and 8 to 10 years, the axial elongations were significantly faster in the post-COVID-19 period than in the pre-COVID-19 period (P = 0.022 and P = 0.005, respectively). However, the rates of axial elongation and myopic progression were not significantly different between pre- and post-COVID-19 in children aged 11 to 15 years (P = 0.065 and P = 0.792, respectively). The average time spent using computers and smartphones and reading time were significantly increased, and the times of physical and outdoor activity were significantly decreased in the post-COVID-19 period compared to the pre-COVID-19 period.

**Conclusions:**

The rates of myopic progression have increased substantially after the spread of COVID-19 with an increase in the home confinement of children. Therefore, it is necessary to control the environmental risk factors for myopia, even in children undergoing treatment for the inhibition of myopic progression.

## Introduction

Myopia is one of the most common ocular disorders worldwide. In addition to genetic susceptibility, several environmental factors, such as prolonged near work, increased digital screen time, and insufficient time spent outdoors, are recognized as important risk factors for myopic progression [1–4]. To control the epidemic of myopia, low-concentration atropine eye drops and specially designed contact lenses and spectacle lenses, such as defocus incorporated multiple segments (DIMS) lenses and aspherical lenslets, are recommended to reduce myopic progression and have now reached high levels of evidence [5–7].

On December 30, 2019, a novel coronavirus disease 2019 (COVID-19) was initially described and rapidly spread worldwide [8]. The World Health Organization (WHO) has declared COVID-19 a “pandemic” outbreak [9]. To control the spread of COVID-19, many countries have locked down their cities in conjunction with the WHO’s recommendations and encouraged people to stay indoors. According to the United Nations Educational, Scientific and Cultural Organization, more than 160 countries have closed schools to limit the spread of COVID-19. This measure involves more than 87% of the world’s student population [10].

In South Korea, schools have replaced all classes with online e-classes in March 2020. Since then, it has been a routine for children to spend most of their time attending online e-classes in front of digital devices. This policy is currently ongoing, inevitably leading to increased screen time, excessive near work, reduced time spent outdoors, and less sunlight exposure for children. As a result, children have been exposed to an environment that is vulnerable to myopic progression during COVID-19. Many parents were concerned about the rapid myopic progression of their children under an unprecedented quarantine. Consequently, many children previously treated with low-concentration atropine have visited our clinic with their anxious parents to control myopic progression despite the confinement under COVID-19.

In this study, we analyzed the rate of myopic progression before and after COVID-19 in children treated with 0.05%, 0.025%, or 0.01% atropine eye drops and classified children according to age (preschool, lower and higher-grade school-aged group). We aimed to evaluate the effect of COVID-19 quarantine on myopic progression.

## Subjects and methods

The medical records of children who visited the pediatric ophthalmology clinic between January 2019 and March 2021 were reviewed. Children aged 5 to 15 years with myopic refraction of at least −1.0 diopter (D) in both eyes and astigmatism of less than 2.5 D were included. This study adhered to the tenets of the Declaration of Helsinki. The study protocol was reviewed and approved by the Institutional Review Board of Eunpyeong St. Mary’s Hospital, part of The Catholic University of Korea College of Medicine (Seoul, Korea; XC20RIDI0175). The institutional review board waived the need for written consent from the participants owing to the retrospective nature of the study. Patient information was anonymized and de-identified prior to analysis.

All children were prescribed low-concentration atropine eye drops before COVID-19 according to the following protocol. The 0.05% atropine eye drops were prescribed when the calculated myopic progression rate exceeded −1.50 D/y. The 0.025% atropine eye drops were prescribed when the calculated myopic progression rate exceeded −1.00 D/y but less than −1.50 D/y. The 0.01% atropine eye drops were prescribed when the calculated myopic progression was below −1.00. The 0.05%, 0.025%, and 0.01% atropine solutions were prepared by diluting 1% atropine eye drop solution (Isopto®Atropine, 10 mg/mL; Alcon, Fort Worth, TX, USA) with 0.9% normal saline. Eye drops were administered daily before bedtime. In Korea, the government restricted outdoor activities from March 2020 and replaced all classes with e-classes instead of attending school. Thus, we defined the setting of the study period as follows. The beginning of the pre-COVID-19 period was set from January 2019 to May 2019, and the endpoint was set in March 2020. The beginning of the post-COVID-19 period was set in March 2020, and the endpoint was set from January 2021 to March 2021. All included patients underwent at least three ophthalmic examinations during the follow-up period. Only children who had a follow-up period of 10 months or more before and after COVID-19 were included in the study. With further analysis of the data, we classified children into preschool-aged (5 to 7 years old), lower grade school-aged (grades 1 to 3, 8 to 10 years old), and higher grade school-aged groups (grade 4 and over, 11 to 15 years old). Patients wearing contact lenses or having other ocular diseases, those undergoing previous ocular trauma/surgery, and those who incorrectly applied eye drops were excluded from the study.

Refractive error was measured using a Huvitz HRK-7000A® auto ref-keratometer (Coburn Technologies, South Windsor, CT, USA) to obtain five reliable results. The results selected for the analyses showed the same measurement values at least three times. Refraction was calculated as the spherical equivalent (SE). Axial length was measured using ocular biometry (IOL Master 500; Carl Zeiss Meditec, Inc. Dublin, CA, USA). Five measurements were obtained for each eye. The axial length was calculated using an automated system included with the equipment. Data from the right eye were used for analysis. In addition, the parents of patients were provided with a questionnaire that included questions concerning the number of hours spent on each of the following activities, such as computer use, smartphone use, reading, physical activities, and outdoor activities throughout the day or week.

All statistical analyses were performed using the Statistical Package for the Social Sciences (SPSS) software version 19.0 (SPSS, Inc., Chicago, IL, USA). The Kolmogorov-Smirnov test was used to evaluate data distributions. The Pearson’s chi-squared test was used to analyze the group difference in categorical data, and Kruskal-Wallis test, paired t-test, and Wilcoxon signed-rank test were used to analyze group difference in continuous data. Multiple linear regression analysis was performed to address the association between the myopic progression and sex, age, initial refractive error, and concentration of atropine eye drops. The statistical significance threshold was set at P = 0.05.

## Results

A total of 103 eyes from 103 children were analyzed in this study. Among these 103 children, 36, 52, and 15 children were included in the 0.05%, 0.025%, and 0.01% atropine groups, respectively. The mean ages were 9.9 years for the 0.05% atropine, 9.8 years for the 0.025% atropine, and 11.7 years for the 0.01% atropine groups. Children in the 0.01% atropine group were older than those in the 0.025% and 0.05% atropine groups (P = 0.041). However, there were no significant differences in sex, SE, axial length, average corneal curvature, anterior chamber depth, central corneal thickness, and lens thickness among the three atropine groups. The mean follow-up periods were 11.4 ± 1.5 months for the pre-COVID-19 period and 11.8 ± 1.7 months for the post-COVID-19 period, showing no significant difference between the two groups (P = 0.228). At baseline, mean SEs were −4.02 ± 2.99 D, −4.33 ± 1.94 D, and −5.62 ± 3.11 D and mean axial lengths were 25.04 ± 1.23 mm, 24.81 ± 1.16 mm, and 25.27 ± 1.71 mm in the 0.05%, 0.025%, and 0.01% atropine groups, respectively (Table 1).

**Table 1:** Baseline demographic characteristics and ocular parameters of study participants.

	Total	0.05% atropine	0.025% atropine	0.01% atropine	*P* value
	(N = 103)	(N = 36)	(N = 52)	(N = 15)	
Age (years)	10.1 ± 2.5	9.9 ± 1.7	9.8 ± 2.6	11.7 ± 3.1	0.041*
Female sex, no. (%)	58 (52.3)	15 (41.7)	28 (53.8)	9 (60.0)	0.387^†^
Spherical equivalent (D)	−4.41 ± 2.56	−4.02 ± 2.99	−4.33 ± 1.94	−5.62 ± 3.11	0.114*
Axial length (mm)	24.96 ± 1.27	25.04 ± 1.23	24.81 ± 1.16	25.27 ± 1.71	0.540*
Average corneal curvature (D)	43.82 ± 1.52	43.51 ± 1.51	43.97 ± 1.48	44.06 ± 1.70	0.246*
Anterior chamber depth (mm)	3.80 ± 0.18	3.83 ± 0.15	3.79 ± 0.19	3.78 ± 0.20	0.253*
Central corneal thickness (mm)	547.88 ± 30.81	549.39 ± 29.90	546.75 ± 31.78	548.20 ± 31.50	0.874*
Lens thickness (mm)	3.35 ± 0.12	3.32 ± 0.12	3.37 ± 0.13	3.38 ± 0.08	0.078*

*P-values calculated using the Kruskal-Wallis test.

^†^*P*-values calculated using the Pearson’s chi-squared test.

Table 2 shows the changes in refractive error and axial length in the 0.05%, 0.025%, and 0.01% atropine groups during pre- and post-COVID-19 periods. A significant myopic progression was observed in the post-COVID-19 period compared to the pre-COVID-19 period in the 0.05% (−0.071 ± 0.053 D/month, −0.039 ± 0.036 D/month, P < 0.001) and 0.025% (−0.068 ± 0.046 D/month, −0.051 ± 0.045 D/month, P = 0.020) atropine groups. In addition, the rates of axial elongation were significantly steeper in the post-COVID-19 period compared to the pre-COVID-19 period in the 0.05% (0.031 ± 0.022 mm/month, 0.025 ± 0.015 mm/month, P = 0.036) and 0.025% (0.030 ± 0.019 mm/month, 0.025 ± 0.016 mm/month, P = 0.026) atropine groups. However, there were no statistically significant differences in the rate of myopic progression and axial elongation between pre- and post-COVID-19 periods in the 0.01% atropine group (P = 0.950 and P = 0.201, respectively).

**Table 2:** Comparisons of the changes of refractive error and axial length between pre- and post-coronavirus disease in the 0.05% atropine, 0.025% atropine, and 0.01% atropine groups.

	Total			0.05% atropine			0.025% atropine			0.01% atropine		
	(N = 103)			(N = 36)			(N = 52)			(N = 15)		
	Pre-COVID-19	Post-COVID-19	*P* value*	Pre-COVID-19	Post-COVID-19	*P* value*	Pre-COVID-19	Post-COVID-19	*P* value*	Pre-COVID-19	Post-COVID-19	*P* value^†^
Δ Spherical equivalent (diopter/month)	−0.047 (0.042)	−0.067 (0.046)	<0.001	−0.039 (0.036)	−0.071 (0.053)	<0.001	−0.051 (0.045)	−0.068 (0.046)	0.020	−0.052 (0.040)	−0.056 (0.031)	0.950
Δ Axial length (mm/month)	0.024 (0.015)	0.030 (0.020)	0.001	0.025 (0.015)	0.031 (0.022)	0.036	0.025 (0.016)	0.030 (0.019)	0.026	0.019 (0.015)	0.023 (0.020)	0.201

Δ = change per month; data are presented as means (standard deviation).

*P-values calculated using the paired t-test.

^†^*P*-values calculated using the Wilcoxon signed-rank test.

**Fig 1:**
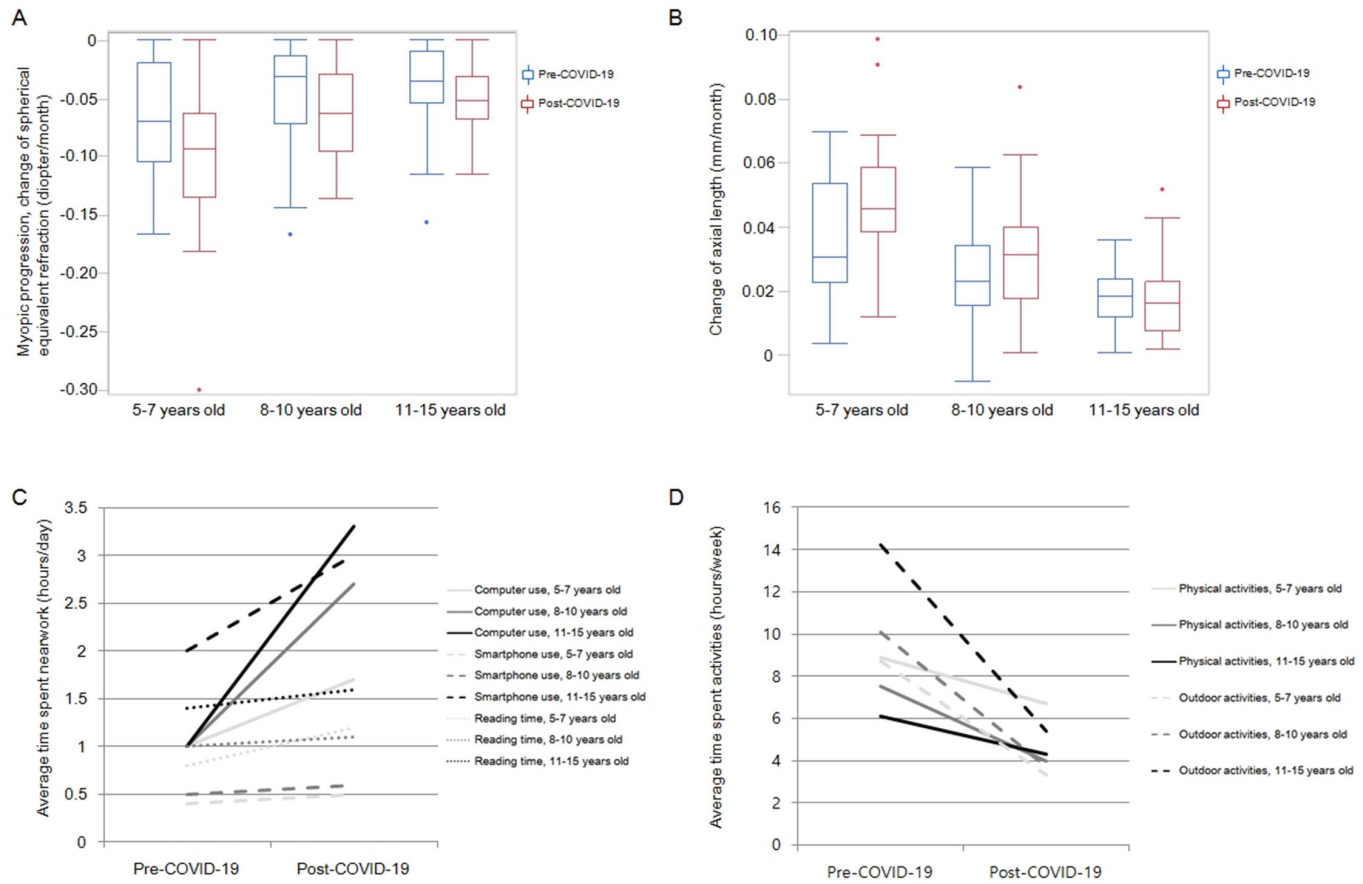
Changes of refractive error, axial length, and questionnaire before and after coronavirus disease 2019 according to children’s age.

We analyzed the changes in refractive error and axial length pre- and post-COVID-19 according to children’s age. Among these 103 children, 23, 42, and 38 children were included in the 5 to 7 years old, 8 to 10 years old, and 11 to 15 years old group, respectively. For children aged 5 to 7 and 8 to 10 years, the mean rates of myopic progression calculated by SE in the post-COVID-19 period were significantly higher than those in the pre-COVID-19 period (P = 0.028 and P = 0.002, respectively). Furthermore, in these age groups, the rates of axial elongation were significantly steeper in the post-COVID-19 period compared with the rates in the pre-COVID-19 period (P = 0.022 and P = 0.005, respectively). However, the rates of myopic progression and axial elongation were not significantly different between pre- and post-COVID-19 periods in children aged 11 to 15 years (P = 0.065 and P = 0.792, respectively) (Table 3 and Fig 1A and 1B). A multiple linear regression analysis revealed that younger age is statistically significant factor to predict the more myopic progression (ß = −0.422, 95% CI, −0.011, −0.005; P < 0.001) and accelerated axial elongation (ß = 0.642, 95% CI, 0.004, 0.006; P < 0.001) in post-COVID-19 period.

**Table 3:** Comparisons of the changes of refractive error and axial length between pre- and post-coronavirus disease 2019 in 5–7, 8–10, and 11–15 years old groups.

	5–7 years old			8–10 years old			11–15 years old		
	(N = 23)			(N = 42)			(N = 38)		
	Pre-COVID-19	Post-COVID-19	*P* value^†^	Pre-COVID-19	Post-COVID-19	*P* value*	Pre-COVID-19	Post-COVID-19	*P* value*
Δ Spherical equivalent (diopter/month)	−0.066 (0.048)	−0.103 (0.064)	0.028	−0.044 (0.041)	−0.064 (0.038)	0.002	−0.038 (0.035)	−0.049 (0.028)	0.065
Δ Axial length (mm/month)	0.036 (0.019)	0.050 (0.019)	0.022	0.024 (0.014)	0.030 (0.018)	0.005	0.017 (0.009)	0.017 (0.011)	0.792

Δ = change per month; data are presented as means (standard deviation).

*P-values calculated using the paired t-test.

^†^*P*-values calculated using the Wilcoxon signed-rank test.

The average time spent indoors, including computer use and smartphone use, was significantly higher in the post-COVID-19 period than in the pre-COVID-19 period in all low-concentration atropine groups (all P < 0.05). On the contrary, the times of physical activity and outdoor activity were significantly lower in the post-COVID-19 period compared to the pre-COVID-19 period in all groups (Table 4) (all P < 0.05). The differences in time spent computer use and outdoor activities between the pre- and post-COVID-19 periods were more prominent in the 8 to 10 years old and 11 to 15 years old groups than in the 5 to 7 years old group (Table 5 and Fig 1C and 1D).

**Table 4:** Questionnaire in the 0.05% atropine, 0.025% atropine, and 0.01% atropine groups.

	Total			0.05% atropine			0.025% atropine			0.01% atropine		
	(N = 103)			(N = 36)			(N = 52)			(N = 15)		
	Pre-COVID-19	Post-COVID-19	*P* value*	Pre-COVID-19	Post-COVID-19	*P* value*	Pre-COVID-19	Post-COVID-19	*P* value*	Pre-COVID 19	Post-COVID-19	*P* value^†^
Computer use (h/day)	1.0 (1.2)	2.7 (1.9)	<0.001	1.1 (1.1)	3.1 (1.9)	<0.001	1.0 (1.4)	2.5 (2.0)	<0.001	0.8 (0.7)	2.7 (1.7)	0.002
Smartphone use (h/day)	1.0 (1.5)	1.5 (2.4)	<0.001	0.7 (0.9)	1.0 (1.6)	0.016	1.1 (1.7)	1.5 (2.4)	0.019	1.6 (1.7)	2.8 (3.7)	0.043
Reading time (h/day)	1.1 (2.1)	1.3 (2.2)	0.013	0.9 (0.6)	1.1 (1.0)	0.155	1.4 (2.9)	1.5 (2.9)	0.115	0.9 (0.6)	1.1 (0.8)	0.102
Physical activity (h/week)	7.3 (6.1)	4.7 (6.2)	<0.001	8.2 (7.2)	4.1 (5.6)	<0.001	7.5 (5.8)	5.7 (7.1)	0.044	4.5 (2.1)	2.6 (1.8)	0.007
Outdoor activity (h/week)	11.3 (11.8)	4.2 (6.1)	<0.001	10.7 (6.7)	3.6 (3.7)	<0.001	13.3 (15.1)	5.0 (7.9)	<0.001	6.2 (4.9)	3.0 (2.6)	0.012

Data are presented as mean (standard deviation).

*P-values calculated using the paired t-test

^†^*P*-values calculated using the Wilcoxon signed-rank test

**Table 5:** Questionnaire according to children’s age.

	5–7 years old			8–10 years old			11–15 years old		
	(N = 23)			(N = 42)			(N = 38)		
	Pre-COVID-19	Post-COVID-19	*P* value^†^	Pre-COVID-19	Post-COVID-19	*P* value*	Pre-COVID-19	Post-COVID-19	*P* value*
Computer use (h/day)	1.0 (1.3)	1.7 (1.8)	0.021	1.0 (1.3)	2.7 (1.7)	<0.001	1.0 (1.0)	3.3 (2.1)	<0.001
Smartphone use (h/day)	0.5 (0.6)	0.6 (0.8)	0.161	0.4 (0.6)	0.6 (0.8)	0.012	2.0 (2.0)	3.0 (3.4)	0.003
Reading time (h/day)	0.8 (0.6)	1.2 (0.7)	0.006	1.0 (0.7)	1.1 (0.8)	0.445	1.4 (3.4)	1.6 (3.4)	0.166
Physical activity (h/week)	8.9 (7.7)	6.7 (8.5)	0.054	7.5 (5.8)	4.0 (3.6)	<0.001	6.1 (5.1)	4.3 (6.6)	0.097
Outdoor activity (h/week)	8.7 (5.0)	3.3 (3.5)	<0.001	10.1 (6.3)	3.7 (4.0)	<0.001	14.2 (17.5)	5.4 (8.7)	<0.001

Data are presented as mean (standard deviation).

*P-values calculated using the paired t-test.

^†^*P*-values calculated using the Wilcoxon signed-rank test.

## Discussion

This study investigated the rate of myopic progression and changes in the lifestyle of children treated with low-concentration atropine before and after the COVID-19 quarantine. Compared to before COVID-19, the myopic progression accelerated after the onset of COVID-19, even in children treated with low-concentration atropine. Myopic progression was most severe in the preschool-aged children, followed by the lower and higher-grade school-aged children. However, the difference in the myopic progression before and after COVID-19 was particularly pronounced in lower-grade school-aged children, and statistically significant changes were also observed in preschool-aged children. The myopic progression also tended to increase in the higher grade school-aged children during the COVID-19 period; however, this failed to reach a level of statistical significance with a relatively unchanged rate of axial elongation between pre- and post-COVID-19 period. In addition, across all ages of children in this study, digital screen time significantly increased, and the time spent on physical and outdoor activities was significantly decreased during the post-COVID-19 period compared to the pre-COVID-19 period. These changes were greater in school-aged children than in preschool-aged children, and more in higher grade than in lower grade school-aged children.

Increased near work and insufficient outdoor activities are considered important risk factors for the incidence and progression of myopia according to several studies [1–4, 11, 12]. A combination of near work through computer uses and reading activity is known to increase the odds of myopia in children aged 9 years (odds ratio [OR]: 1.072, 95% confidence interval [CI]: 1.047–1.098) [13]. In addition, time spent using a smartphone was independently associated with myopia in a study of 418 students (OR: 1.08, 95% CI: 1.03–1.14) [14]. The outbreak of COVID-19 has made it inevitable to hold all classes online and make children stay indoors. As a result, children are forced to use digital devices excessively under COVID-19 quarantine compared to the pre-COVID-19 period. Increased time spent indoors, close contact with computers and smartphones, and restricted outdoor and physical activities could potentially be related to myopic progression during the COVID-19 pandemic outbreak. The increased risk of myopia onset and progression may vary according to ethnicity (Asian, Caucasian, Hispanic, and African American). However, pandemic-associated lockdown may affect the incidence and progression of myopia globally. Therefore, it is necessary to monitor children’s use of electronic devices (computers, tablets, and smartphones) and to promote their outdoor activities to prevent myopic progression worldwide.

In a study by Sumitha et al. [15], the proportion of myopia diagnosis in the pediatric ophthalmic outpatient department was 63% in March and April 2020, similar to the proportion of 64% in March and April 2019. However, the authors concluded that a long-term change in the environment under COVID-19 might lead to an increased incidence of myopia and myopic progression in children over a long period. In fact, in another study conducted between June 2019 and June 2020, researchers have reported that the half-year incidence rate of myopia increased from 8.5% in the pre-COVID-19 period to 13.6% in the post-COVID-19 period [16].

In this study, there were significant differences in the change in mean SE and axial length between the pre- and post-COVID-19 periods, especially in the lower grade school-aged children. In addition, the degree of absolute myopic progression was greater in preschool-aged children than in lower and higher-grade school-aged children. Also, the overall time spent on digital devices was longer in higher grade school-aged children than in preschool-aged and lower grade school-age children. However, the rate of axial elongation was relatively unchanged during the COVID-19 period in higher grade school-aged children group compared to the other groups. In addition, we found that the younger the children, the myopic progression and axial elongation accelerated in the post-COVID-19 period in the multivariate linear regression analysis adjusted sex, initial refractive error, and different low-concentration atropine eye drops as covariates. We concluded that younger children are more vulnerable to environmental risk factors associated with myopic progression than older children. Our results were consistent with the findings of Wang et al. [17], who reported that noticeable myopic progression was found in younger children aged 6 to 8 years during the COVID-19 pandemic. However, the previous study did not include preschool-aged children, simultaneous measurement of time spent indoors and outdoors, and information on ocular biometry.

Atropine, a nonselective muscarinic acetylcholine receptor antagonist, has been reported to slow down myopic progression in children [18]. Different concentrations and various doses of atropine have been widely used topically as eye drops in many countries with diverse ethnicities [19–24]. Photophobia is the most common cause of atropine eye drop discontinuation. The incidence rates of photophobia are 6.3%, 17.8%, and 43.1% in low-dose atropine (0.01%), moderate-dose atropine (>0.01% to < 0.5%), and high-dose atropine (0.5% to 1.0%), respectively [25]. Fortunately, because the intensity of light indoors was relatively low compared to that of light outdoors, a low-concentration atropine eye drop was well tolerated by children with home confinement under COVID-19. However, considering that the rate of myopic progression in the post-COVID-19 period was higher than that in the pre-COVID-19 period despite the use of low-concentration atropine eye drops, more rigorous and comprehensive age-based myopia control strategies are needed during the quarantine period.

This study has some limitations. First, children without atropine eye drop treatment were excluded. This is because axial length was not routinely performed during clinical examinations in children without atropine treatment. However, in a previous study, we found that changes in refraction and axial length in normal controls were −0.134 D/month and 0.046 mm/month, respectively [26]. Compared to these previous results, changes in refraction and axial length in the present study were relatively lower under low-concentration atropine treatment both in the pre- and post-COVID-19 periods. Second, refraction results were obtained without cycloplegia. However, axial length using ocular biometry was examined in children, with an average of 5 readings within a deviation of 0.05 mm or less, and the results supported the analysis of refractive changes.

In summary, the rate of myopic progression accelerated after the COVID-19 period compared to the pre-COVID-19 period in children treated with low-concentration atropine, especially in younger children. It is estimated that the quarantine may have affected the myopic progression under COVID-19. Therefore, it is necessary to control the environmental risk factors for myopia, even in children undergoing treatment for the inhibition of myopic progression.

## Supporting information

S1 File (DOC)

S2 File (DOC)

S1 Raw data (XLS)
