# Supplementary material for: Influence of coronavirus disease 2019 on myopic progression in children treated with low-concentration atropine
Source: PLoS One. 2021 Sep 14;16(9):e0257480. doi: 10.1371/journal.pone.0257480 (PMC8439482; doi:10.1371/journal.pone.0257480)
Supplement: S1 File — (DOC) [file pone.0257480.s001.doc]

**Questionnaire about the lifestyle in pre-COVID-19 and post-COVID-19 period**

We would like to investigate your child's lifestyle before and after the COVID-19 and utilize it to study the impact of COVID-19 on the progression of myopia. All information will be anonymized and de-identified prior to analysis. Please remember and consider the lifestyle of your child in the year before COVID-19 and in the year after COVID-19, and fill out the questionnaire below accurately.

Name:

Date:

|  | Before COVID-19 | After COVID-19 |
| --- | --- | --- |
| 1. Computer use (per day) | hour minute/day | hour minute/day |
| 1. Smartphone use (per day) | hour minute/day | hour minute/day |
| 1. Reading time (per day) | hour minute/day | hour minute/day |
| 1. Physical activity (per week) | hour minute/week | hour minute/week |
| 1. Outdoor activity (per week) | hour minute/week | hour minute/week |
