# Supplementary material for: Influence of coronavirus disease 2019 on myopic progression in children treated with low-concentration atropine
Source: PLoS One. 2021 Sep 14;16(9):e0257480. doi: 10.1371/journal.pone.0257480 (PMC8439482; doi:10.1371/journal.pone.0257480)
Supplement: S2 File — (DOC) [file pone.0257480.s002.doc]

**코로나 시기 전/후의 생활패턴의 변화**

코로나 시기 전 후의 귀하의 아동의 생활 패턴을 조사하여 앞으로 현재의 근시 진행 억제 치료에 있어 활용을 하고자 합니다.

바쁘신 와중에도 아동의 생활 패턴에 대해 고민하시고 정확하게 작성해 주시면 감사하겠습니다.

아이 이름:

작성 날짜:

| 항 목 | 코로나 전 | 코로나 후 |
| --- | --- | --- |
| 1. 하루 중 **컴퓨터/태블릿** 사용이 몇 시간 몇 분? | 시간 분/1일 | 시간 분/1일 |
| 1. 하루 중 **핸드폰** 사용이 몇 시간 몇 분? | 시간 분/1일 | 시간 분/1일 |
| 1. 하루 중 **독서 시간**은 몇 시간 몇 분? | 시간 분/1일 | 시간 분/1일 |
| 1. 일주일 중 **체육활동**이 몇 시간 몇 분? (**실내/실외포함**) | 시간 분/1주 | 시간 분/1주 |
| 1. 일주일 중 **야외활동**이 몇 시간 몇 분? | 시간 분/1주 | 시간 분/1주 |
